# The Association between Vigorous Physical Activity and Stress in Adolescents with Asthma

**DOI:** 10.3390/ijerph18073467

**Published:** 2021-03-26

**Authors:** Sunga Kong, Wi-Young So, Seyong Jang

**Affiliations:** 1 Department of Clinical Research Design and Evaluation, SAIHST, Sungkyunkwan University, Seoul 06351, Korea; sunga00kong@gmail.com; 2 Samsung Medical Center, Patient-Centered Outcomes Research Institute, Seoul 06351, Korea; 3 Sports Medicine Major, College of Humanities and Arts, Korea National University of Transportation, Chungju 27469, Korea; wowso@ut.ac.kr; 4 Department of Taekwondo, College of Arts and Physical Education, Gachon University, Seongnam 13120, Korea

**Keywords:** adolescents, asthma, exercise, stress, vigorous physical activity

## Abstract

Asthma is a very common condition that affects 5–10% of the global population, and its prevalence is increasing. Vigorous physical activity (PA) is effective in improving cardiorespiratory fitness and managing stress. This study aimed to investigate the association between vigorous PA and stress among Korean adolescents with asthma using large-scale survey data. The questionnaire data of 57,303 adolescents were analyzed using raw data from the 2019 Korea Youth Risk Behavior Web-Based Survey. We performed logistic regression analysis to calculate the stress odds ratios (ORs) and 95% confidence intervals (CIs) for asthma and non-asthma groups using models 1 and 2. We also performed logistic regression analysis to calculate the stress OR for the asthma group with vigorous PA and non-vigorous PA using models 1, 2, and 3. Model 1 was adjusted for age, sex, obesity, smoking, and alcohol status; model 2 was further adjusted for household income, academic achievement, and comorbidities. Model 3 was further adjusted for moderate activity and resistance exercise. The OR of stress was 20% higher in the asthma group than in the non-asthma group (*p* < 0.05). In the fully adjusted models, the OR for the non-asthma group with vigorous PA versus non-vigorous PA was 0.89 (95% CI: 0.84–0.94). However, the OR for the asthma group with vigorous PA versus non-vigorous PA was 0.70 (95% CI: 0.57–0.86), indicating that adolescents who engage in vigorous PA had lower stress in the asthma group (*p* < 0.05). This study demonstrated that adolescents with asthma had higher stress levels than those without asthma; however, vigorous PA was associated with lower stress. These effects were more pronounced in adolescents with asthma.

## 1. Introduction

Asthma is a very common condition that affects 5–10% of the global population [1], and its prevalence is increasing [2]. The prevalence of asthma among adolescents in the Republic of Korea increased from 2.7% in 1995 to 7.5% in 2010 [3]. Asthma is a chronic respiratory disease that is characterized by airway hyper-responsiveness, inflammation, and airflow obstruction [4], all of which result in higher oxidative stress [5]. Many previous studies have demonstrated a link between asthma and mental health problems [6,7]. Stress has been associated with increased asthma prevalence in multiple studies of adults and children [8]. Stress can influence health beliefs and behaviors that may affect asthma management [9]. Therefore, asthma management must incorporate multiple factors.

A stress management model is needed for asthma management [10]. Asthma that is associated with mental health problems leads to more frequent healthcare utilization by these patients, increasing the healthcare requirement for this condition [11]. The management of stress—including the burden of psychological distress—has been recognized as an important health determinant for both adults and adolescents [11,12]. According to Statistics Korea, in 2018, the intensity of stress for Korean adolescents was as high as 39.8% in 2017, and 4 out of 10 Korean adolescents still experience high levels of stress [13]. Such exposure to stress can be particularly harmful for adolescents because they are highly vulnerable to the effects of chronic stress at this sensitive and critical period of adolescent development [14]. Therefore, the management of stress for adolescents with asthma should be a priority along with mental healthcare.

There are several stress management programs; however, physical activity (PA) provides specific psychological treatment that may be particularly effective for patients for whom more conventional psychological interventions are less appropriate [15]. Furthermore, PA as a modifiable factor is reported to be the strongest variable associated with respiratory disease and greatly affects physiological age, dyspnea, airflow obstruction index, body mass index (BMI), airway obstruction, dyspnea, exercise capacity index, forced expiratory volume in one second % (FEV_1_), and 6-min walk distance [16]. Previous research has indicated that PA has a positive effect on mental health and improves asthmatic symptoms in patients with asthma. Furthermore, the amount of sedentary time has been found to influence the prevalence of asthma in Korean adolescents [17].

However, previous studies on the management of asthma used small sample sizes, and the sample population was usually limited to adult [18] or pediatric patients [19,20]. Studies on the association between exercise, such as vigorous PA, and stress among adolescents with asthma are unavailable [21]. Vigorous PA is particularly more effective in strengthening cardiorespiratory fitness [22] compared with moderate activity; however, no study has confirmed its effect by evaluating vigorous PA in adolescents. Additionally, in this field, large-scale research is necessary for proactive healthcare for adolescents [23]. Thus, this study analyzed the association between vigorous PA and stress in adolescents with asthma using the data from a large-scale survey of Korean adolescents.

## 2. Materials and Methods

### 2.1. Participants

This cross-sectional study used raw data from the 2019 Korea Youth Risk Behavior Web-Based Survey (KYRBWS)-15^th^, which was a self-administered, online, statistical survey that was approved by the Korean government to understand the health behavior of Korean middle and high school students (7th–12th grade). Of the total 60,100 survey participants, 57,303 (95.3%) were analyzed in the current study. Since private or identifiable information such as telephone numbers, social security numbers, and home addresses was not collected by the KYRBWS-XV, ethical approval was not required for this study. Furthermore, all research procedures were controlled and approved by the Korea Centers for Disease Control and Prevention (KCDCP) and conducted in accordance with the principles outlined in the Declaration of Helsinki. All the details pertaining to data collection procedures have been reported by the KCDCP [24], and the reliability and validity of the KYRBWS questionnaires have been evaluated [25,26].

### 2.2. Definition of Groups

If the participant responded “yes” to the question “Did a doctor ever say that you had asthma?”, this participant was allocated to the asthma group. If the participants responded “no” or “don’t know”, they were allocated to the non-asthma group. Participants were assigned to the non-vigorous PA group if they responded “no exercise”, “1 day”, or “2 days” to the question “In the last 7 days, how many days did you do vigorous PA that caused you to be out of breath or sweat for more than 20 min?” Participants were allocated to the vigorous PA group if they answered “3 days”, “4 days”, or “more than 5 days” to this question [27].

### 2.3. Main Outcome

Participants who responded “very often,” “often,” or “seldom” to the question “How often do you feel stress?” were assigned to the stress category, and those who answered “very seldom” or “never” were assigned to the non-stress category.

### 2.4. Covariate Variables

Sociodemographic characteristics included age, sex, BMI, and grade (middle or high school). Smoking status was classified using the responses “no” and “yes” to the question “Have you ever smoked (even once) in the past?” Alcohol consumption was classified using the responses “no” and “yes” to the question “Have you ever drunk more than one glass of alcohol?”

Economic status included household income and was classified as “high,” “medium high,” “medium,” “medium low,” or “low” based on the response to the question “How would you classify your family’s economic status?” Academic achievement was classified as “high,” “medium high,” “medium,” “medium low,” or “low” based on the response to the question “How was your academic achievement over the past 12 months?”

Health-related PA included moderate activity and resistance exercise. Moderate activity was classified using the responses “no exercise,” “1 day,” “2 days,” “3 days,” “4 days,” or “more than 5 days” to the question “In the last 7 days, on how many days did you do PA (regardless of type) for more than 60 min that resulted in an accelerated heart rate or feeling out of breath?” Resistance exercise was classified using the responses “no resistance exercise,” “1 day,” “2 days,” “3 days,” “4 days,” or “more than 5 days” to the question “In the last 7 days, on how many days did you do resistance exercises such as push-ups, sit-ups, lifting dumbbells and barbells, chin-ups, or use of parallel bars?”

### 2.5. Statistical Analyses

All analyses were conducted using the STATA software, version 15.0 (STATA Corp., College Station, TX, USA). All variables were expressed as mean, standard deviation, and percentages (%). The characteristics of the groups (non-asthma and asthma) were compared using an independent *t*-test and a Chi-square test. These tests were conducted to analyze different physical characteristics, smoking status, alcohol consumption, household income, academic achievement, moderate activity, vigorous PA, and resistance exercise in relation to asthma. The stress odds ratios (ORs) (95% confidence interval (CI)) according to the frequency of vigorous PA was calculated using logistic regression. The stress OR according to the asthma group and the vigorous PA group was calculated using multiple logistic regression with models 1 and 2. The OR (95% CI) of stress according to vigorous PA versus non-vigorous PA in the asthma and non-asthma groups was calculated using multiple logistic regression with models 1, 2, and 3. Model 1 was adjusted for age, sex, and obesity (BMI of <18.5, 18.5–22.9, and ≤23 km/m^2^); model 2 was further adjusted for household income (<medium-high and ≥medium-high), academic achievement (<medium-high and ≥medium-high), and comorbidities (atopic dermatitis or allergic rhinitis); and model 3 was further adjusted for resistance exercise (<3 days per week or ≥3 days per week) and moderate activity (<5 days per week or ≥5 days per week). All statistical significance was set at *p* < 0.05.

## 3. Results

The characteristics of the asthma and non-asthma groups are shown in Table 1. Of the total 57,303 participants, 4020 (7.02%) were assigned to the asthma group. The asthma group was older, had a 5.5% higher male-to-female ratio, and had a 0.6 kg/m^2^ higher BMI than the non-asthma group (*p* < 0.001). Academic achievement was better in the asthma group than in the non-asthma group (*p* < 0.001). Moderate activity and resistance exercise were significantly higher in the asthma group (*p* < 0.05). No significant differences in vigorous PA were found between the groups (*p* > 0.05) (Table 1).

**Figure 1:**
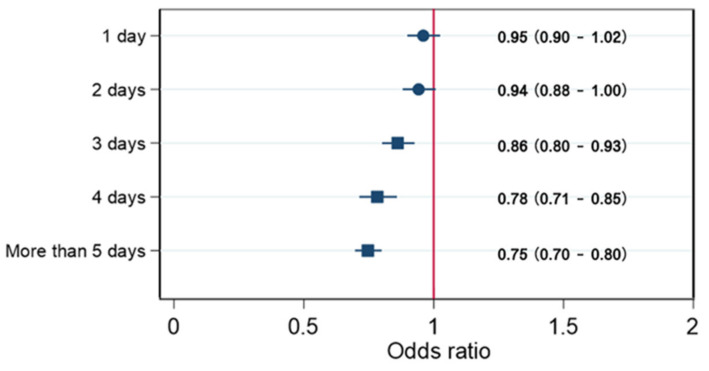
Odds ratios (95% confidence interval (CI)) of stress in relation to vigorous physical activity.

iLogistic regression was used to determine the relationship of stress with the frequency of vigorous PA. The OR was significantly decreased when vigorous PA was performed for more than three days a week, 0.87 (0.82–0.93); more than four days a week, 0.79 (0.72–0.87); and more than five days a week, 0.76 (0.71–0.81) (*p* < 0.05) (Figure 1).

This figure shows the OR (95% CI) of stress in relation to vigorous physical activity, adjusted for age, sex, obesity (BMI of <18.5, 18.5–22.9, and ≤23 kg/m^2^), smoking status, and alcohol status. 

In relation to stress, the asthma group had an OR of 1.24 (1.14–1.36) in model 1 and an OR of 1.14 (1.04–1.25) in model 2. This group had a higher OR than the non-asthma group (*p* < 0.05). Conversely, the vigorous PA group had an OR of 0.82 (0.78–0.86) in model 1 and an OR of 0.84 (0.80–0.88) in model 2. The OR of this group had a lower OR than that of the non-vigorous PA group (*p* < 0.05) (Table 2).

The non-asthma group that engaged in vigorous PA displayed an OR of 0.82 (0.79–0.86) in model 1, an OR of 0.84 (0.80–0.88) in model 2, and an OR of 0.89 (0.84–0.94) in model 3. This group had a lower OR than the non-vigorous PA group. The asthma group with vigorous PA had an OR of 0.77 (0.64–0.92) in model 1, an OR of 0.78 (0.65–0.93) in model 2, and an OR of 0.70 (0.57–0.86) in model 3. This group had a lower OR than the non-vigorous PA group (*p* < 0.05) (Table 3).

## 4. Discussion

This study used data from a large-scale survey of Korean adolescents and revealed that adolescents with asthma had higher stress levels than those without asthma. However, more than 20 min of vigorous PA for more than three days a week resulted in lower stress among these participants. Vigorous PA was related to low levels of stress, but adolescents with asthma experienced lower stress than those without asthma.

In this study, adolescents with asthma displayed 1.24 and 1.14-fold higher stress levels than adolescents without asthma following adjustments in models 1 and 2, respectively. These results were consistent with the results of previous studies [28,29]. A previous study reported that emotional stress can promote or exacerbate acute and chronic asthma [29]. Another study that followed-up 326 adults for over a year revealed that higher perceived stress is correlated with worse asthma control and poor quality of life [30]. Since 39.8% of Korean adolescents reported a perception of stress in 2017 [13], customized health management for adolescents with asthma is necessary because adolescents with asthma are more vulnerable to stress than those without asthma.

In this study, the OR of stress in relation to the frequency of vigorous PA was identified in order to examine the association between vigorous PA and lower levels of stress among adolescents. Several studies have demonstrated a strong correlation between stress and PA among healthy adolescents [31,32,33]. However, the effect of PA on stress varies according to the intensity of the exercise. Moderate activity alone was also effective in reducing stress among adolescents [31]; however, these results were not consistent with those for light physical activity [34]. Stress affects physical and mental health and can cause distress-related depression and suicide, difficulty in sleeping, cardiovascular disease, and immune system dysfunction. Adolescents with mental disorders can have severe social and occupational dysfunction in adulthood [35]. Many studies have demonstrated the positive effect of exercise and PA on mental health [31,32,33,34,35,36]. Furthermore, our study demonstrated that >20 min of vigorous PA for more than three times a week was related to low stress levels among adolescents.

Finally, our study detected that regular vigorous PA, despite higher stress levels in the asthma group, had a lower impact on adolescents with asthma than on those without asthma. These results are consistent with the results of previous studies. Adolescent PA guidelines recommend more than 60 min of daily moderate-to-vigorous PA with additional vigorous PA more than three times per week [37]. Our results indicated that vigorous PA was an independent factor that lowered stress among adolescents with asthma after adjusting for exercise behavior. Oxidative stress is recognized as a burden in patients with asthma and is one of the risk factors that induces and exacerbates respiratory diseases, such as atherosclerosis, cardiovascular disease, chronic obstructive pulmonary disease, and bronchial asthma [38,39,40]. Further, oxidative stress is linked to emotional stress [41]. However, there is evidence that exercise increases antioxidant protection among adolescents [42]. Structured exercise programs were found to result in better lung function in asthmatic children, potentially due to the antioxidant effect of PA [20]. Our results cannot suggest that regular vigorous PA actually had an antioxidant effect on adolescents; however, clinically, progressive vigorous PA will be helpful in reinforcing cardiorespiratory function in adolescents [27], reducing oxidative stress [43], and improving physical and psychological stress [44]. Our results, although specifically for adolescents with asthma, confirmed that regular vigorous PA is associated with less stress than irregular PA, despite the fact that the cause is not clear. However, physical fitness and the maintenance of healthy body weight may actually reduce the symptoms of asthma and could help control the disease [45,46]. Furthermore, fitness levels, including cardiorespiratory fitness, among adolescents with asthma are lower than those among adolescents without asthma [47]. In addition to encouraging simple physical activity, a program to improve cardiopulmonary function through high-intensity exercise is needed.

This study had several limitations. First, this study was based on a questionnaire survey; therefore, a history of asthma treatment and severity could not be determined. Additionally, some participants may not have been clinically diagnosed but still may have experienced some symptoms of asthma. However, the incidence rate of asthma among Korean adolescents is approximately 7%, and this sample demonstrated a prevalence of 7.05%, which was similar to the domestic criteria. Second, this study was conducted online using multiple-choice questions; therefore, it is possible that the exact amount of vigorous PA and self-reported PA may have varied. However, our data identified moderate and resistance strengthening exercises, thus enabling the classification of the intensity and type of exercise. Third, racial and socioeconomic differences may have existed, and these factors were not considered in this study. Fourth, this study was a cross-sectional study with a retrospective cohort design. Therefore, we did not examine cause and effect—only interrelationships. Nevertheless, this study had the advantage of being a government-oriented large survey with 57,303 participants.

## 5. Conclusions

In this study, stress was higher among adolescents with asthma than adolescents without asthma. However, more than 20 min of vigorous PA more than three times a week was demonstrated to lower stress, and the association between stress and vigorous PA was more pronounced for adolescents with asthma than those without asthma. It is therefore necessary to emphasize the importance of vigorous PA for adolescents with elevated stress along with respiratory diseases such as asthma.

## Figures and Tables

**Table 1:** Characteristics of participants with asthma and without asthma.

Variables.	Total (*n* = 57,303)	Asthma (*n* = 4020)	Non-Asthma (*n* = 53,283)	*p*
Age (year)	15.0 (1.8)	15.1 (1.7)	15.0 (1.8)	<0.001 ***
Male, N (%)	29,841 (52.1)	2300 (57.2)	27,541 (51.7)	<0.001 ***
Body mass index (kg/m^2^)	21.4 (3.6)	21.9 (3.7)	21.3 (3.5)	<0.001 ***
Grade (%)				<0.001 ***
Middle school	51.3	47.4	51.6	
High school	48.7	52.6	48.4	
Smoking status (%)				<0.001 ***
No	87.7	84.3	89.9	
Yes	12.3	15.7	12.1	
Alcohol consumption (%)				<0.001 ***
No	61.2	56.6	61.5	
Yes	38.8	43.4	38.5	
Household income (%)				0.281
High or medium-high	39.3	40.1	39.2	
Medium, medium-low, or low	60.7	59.9	60.8	
Academic achievement (%)				0.001 **
High or medium-high	38.3	40.7	38.1	
Medium, medium-low, or low	61.7	59.3	61.9	
Moderate physical activity (%)				0.021 *
<5 times per week	15.2	83.4	84.8	
≥5 times per week	84.8	16.6	15.2	
Vigorous physical activity (%)				0.064
<3 days per week	67.1	65.8	67.2	
≥3 days per week	32.9	34.2	32.8	
Resistance exercise (%)				0.004 **
None or once per week	67.3	65.2	67.4	
≥2 times per week	32.7	34.8	32.6	

Data are presented as mean ± standard deviation. * *p* < 0.05, ** *p* < 0.01, *** *p* < 0.001; tested by independent *t*-test.

**Table 2:** Odds ratios of stress status in relation to asthma or vigorous physical activity.

Variables.	Non-Asthma	Asthma	*p*	Non-Vigorous Physical Activity	Vigorous Physical Activity	*p*
Stress						
^a^ Model 1 OR (95% CI)	Reference	1.24 (1.14–1.36)	<0.001 ***	Reference	0.82 (0.78–0.86)	<0.001 ***
^b^ Model 2 OR (95% CI)	Reference	1.14 (1.04–1.25)	0.004 **	Reference	0.84 (0.80–0.88)	<0.001 ***

OR: Odd ratio; CI: confidence interval; BMI: body mass index. ** *p* < 0.01, *** *p* < 0.001; tested by multiple logistic regression analysis. ^a^ Model 1: adjusted for age, sex, obesity (BMI of <18.5, 18.5–22.9, and ≤23 kg/m^2^), smoking status, and alcohol consumption. ^b^ Model 2: further adjusted for household income (<medium-high and ≥medium-high), academic achievement (<medium-high and ≥medium-high), and comorbidities (atopic dermatitis or allergic rhinitis).

**Table 3:** Odds ratios of stress status in relation to asthma and vigorous physical activity.

Variables.	Non-Asthma Non-Vigorous	Non-Asthma Vigorous	*p*	Asthma Non-Vigorous Physical Activity	Asthma Vigorous Physical Activity	*p*
Stress						
^a^ Model 1 OR (95% CI)	Reference	0.82 (0.79–0.86)	<0.001 ***	Reference	0.77 (0.64–0.92)	0.005 **
^b^ Model 2 OR (95% CI)	Reference	0.84 (0.80–0.88)	<0.001 ***	Reference	0.78 (0.65–0.93)	0.006 **
^c^ Model 3 OR (95% CI)	Reference	0.89 (0.84–0.94)	<0.001 ***	Reference	0.70 (0.57–0.86)	0.001 **

OR: Odd ratio; CI: confidence interval; BMI: body mass index. ** *p* < 0.01, *** *p* < 0.001; tested by multiple logistic regression analysis. ^a^ Model 1: adjusted for age, sex, obesity (BMI of <18.5, 18.5–22.9, and ≤23 kg/m^2^), smoking status, and alcohol consumption. ^b^ Model 2: further adjusted for household income (<medium-high and ≥medium-high), academic achievement (<medium-high and ≥medium-high), and comorbidities (atopic dermatitis or allergic rhinitis). ^c^ Model 3: further adjusted for resistance exercise (yes or no; yes = 3 or more time per week) and moderate activity (yes or no; yes = 5 or more time per week).

## Data Availability

The data that support the findings of this study are openly available in Korea Centers for Disease Control and Prevention in May 25, 2020 at http://yhs.cdc.go.kr/.
